# Phenotypic plasticity in life‐history traits of *Daphnia galeata* in response to temperature – a comparison across clonal lineages separated in time

**DOI:** 10.1002/ece3.1924

**Published:** 2016-02-22

**Authors:** Nicole Henning‐Lucass, Mathilde Cordellier, Bruno Streit, Klaus Schwenk

**Affiliations:** ^1^ Biodiversity and Climate Research CentreGeorg‐Voigt‐Straße 14‐1660325Frankfurt/MainGermany; ^2^ Institute for Environmental SciencesUniversity Koblenz‐LandauFortstraße 776829Landau in der PfalzGermany; ^3^ University of Hamburg, Biozentrum GrindelMartin‐Luther‐King Platz 320146HamburgGermany; ^4^ Department of Ecology and EvolutionFrankfurt UniversityMax‐von‐Laue‐Straße 1360438Frankfurt am MainGermany

**Keywords:** Climate change, *Daphnia*, freshwater ecosystems, life‐history traits, micro‐evolution, resurrection ecology

## Abstract

Climatic changes are projected to result in rapid adaptive events with considerable phenotypic shifts. In order to reconstruct the impact of increased mean water temperatures during past decades and to reveal possible thermal micro‐evolution, we applied a resurrection ecology approach using dormant eggs of the freshwater keystone species *Daphnia galeata*. To this end, we compared the adaptive response of *D. galeata* clones from Lake Constance of two different time periods, 1965–1974 (“historical”) versus 2000–2009 (“recent”), to experimentally increased temperature regimes. In order to distinguish between genetic versus environmentally induced effects, we performed a common garden experiment in a flow‐through system and measured variation in life‐history traits. Experimental thermal regimes were chosen according to natural temperature conditions during the reproductive period of *D. galeata* in Central European lakes, with one additional temperature regime exceeding the currently observable maximum (+2°C). Increased water temperatures were shown to significantly affect measured life‐history traits, and significant “temperature × clonal age” interactions were revealed. Compared to historical clones, recent clonal lineages exhibited a shorter time to first reproduction and a higher survival rate, which may suggest temperature‐driven micro‐evolution over time but does not allow an explicit conclusion on the adaptive nature of such responses.

## Introduction

According to the Intergovernmental Panel on Climate Change, the globally averaged combined land and ocean surface temperature data show a warming of 0.85°C over the period 1880–2012. A further increase of the global mean surface temperature by the end of the 21st century (relative to 1986–2005) is projected to range from 0.3 to 4.8°C, depending on the choice of emission scenario (IPCC, 2014). Rising temperatures have already affected the biota from organism to ecosystem levels (as reviewed in Walther et al. 2002; Parmesan 2006). Depending on species‐specific physiological limitations and evolutionary potential, global warming may lead to a change in species distribution (Hampe 2004; Heino et al. 2009; Wittmann et. al 2016), or assuming that dispersal is restricted, to either local extinction or local persistence through phenotypic plasticity or micro‐evolutionary adaptation (Bellard et al. 2012). Genetic changes through recombination and selection may occur quickly and result in a rapid evolutionary response of natural populations to current global warming (Salamin et al. 2010; Hoffmann and Sgro 2011).

Freshwater ecosystems are especially vulnerable to climatic changes (Allan et al. 2005; Woodward et al. 2010), as the internal temperature and metabolic rates of the predominantly ectothermic taxa are directly linked to environmental temperatures (Dupuis and Hann 2009; Wagner and Adrian 2011). Several temperature effect studies focus on the small crustacean *Daphnia*, which is affected in its swimming behavior (Ziarek et al. 2011), chemical signaling (Lass and Spaak 2003), growth and reproduction rates (Rinke and Petzoldt 2003), and other parameters. Besides playing an important ecological role in freshwater ecosystems as a planktonic grazer with a central position in lake food webs (Carpenter et al. 2001; Lampert and Sommer 2007), *Daphnia* has an advantageous mode of reproduction for studying adaptation processes. *Daphnia* females are cyclic parthenogens that reproduce asexually under benign environmental conditions, producing populations consisting of clonal lineages with multiple copies of the same genotype (Hebert and Crease 1983; Ebert 2005). Under stressful conditions, such as extreme temperatures or food scarcity, females switch to sexual reproduction, producing encapsulated dormant eggs, the majority of which sink down in the water column and become covered by lake sediment, forming a resting egg bank (Hairston 1996). Dormant eggs remain viable in the sediment for years and decades, in extreme cases up to several centuries (Frisch et al. 2014). Resurrection ecology approaches make use of these archives by hatching dormant eggs extracted from dated sediment layers (Jankowski and Straile 2003; Kerfoot and Weider 2004). The possibility to perform genetic or life‐history experiments with “historical” clones allows to test for micro‐evolutionary responses to variation in environmental parameters, as exemplified in Hairston et al. (2001), Decaestecker et al. (2007) and Orsini et al. (2013).

Evidence for micro‐evolutionary responses to an increase in temperature under experimental conditions in *Daphnia* species was recently documented in a study by Geerts et al. (2015). The authors used a resurrection ecology approach to quantify evolutionary changes in the critical thermal maximum (CT_Max_), an index of heat tolerance, comparing CT_Max_ values of historical and recent *Daphnia magna* clones. Recent clones and clones exposed to higher temperatures showed an increase in CT_Max_, suggesting that *Daphnia* has the potential to evolve higher heat tolerances. In earlier studies, van Doorslaer et al. (2009a, 2010) revealed thermal genetic adaptation in outdoor mesocosm, as well as indoor laboratory selection experiments followed by common garden experiments to quantify micro‐evolutionary changes. Rapid genetic changes were observed upon exposure to different temperature regimes, affecting size at maturity and intrinsic growth rates in *Daphnia* (see also de Meester et al. 2011).

In the present study, we applied a resurrection ecology approach using the genetic archive of natural populations to assess the evolutionary adaptive potential of *Daphnia galeata*, a species particularly common in Holarctic temperate lakes (Brooks 1957). As previously observed, changes in CT_Max_ are potentially a result of changes in the maximum rather than average temperature (Geerts et al. 2015). We combined our resurrection experiment with indoor laboratory common garden experiments, comparing life‐history responses of clonal lineages resurrected from two different time periods subjected to increased temperatures. The annual mean water temperature (recorded at 0.5 m depth) of Lake Constance increased approximately 0.03°C per year from 1965 to 2010 (KLIWA, 2011). Upper water layers, which are inhabited by *D. galeata*, are more strongly affected and the duration as well as temperature of the warmer summer period increased in Lake Constance during the last decades (KLIWA, 2011). While the typical mean water temperature in upper water layers was in the range between 20 and 22°C during the warmer summer months from 2000 to 2010, the temperature means in upper water layers during summer between 1965 and 1975 were about 18–20°C (Stich and Lampert 1981). Therefore, we hypothesized that under elevated temperature regimes “recent” clones of *D. galeata* would exhibit a higher fitness than “historical” lineages.

## Materials and Methods

### Sampling & hatching

In total, 12 sediment cores were sampled from the bottom of Lake Constance, a mesotrophic, monomictic freshwater lake on the border between Germany, Switzerland, and Austria. Four cores at a time were collected in December 2002, September 2004, and 2009 at 180–220 m depth near the Langenargener Bucht (47°37′21″N, 9°26′24″E). Sediment sampling was solely carried out at deeper water sites as density of dormant propagules is highest in the deepest parts of a lake due to lateral transports (Carvalho and Wolf 1989). After sampling, sediment cores were wrapped in aluminum foil and sealed to prevent premature exposure to light and oxygen. Subsequently, sediment cores were sliced in a laboratory and samples were stored in opaque sealed containers at 4°C (i.e., the bottom temperature at sampling sites) to avoid hatching stimuli. Afterward, the sediment was dated via annual lamination counting (Viitasalo and Katajisto 1994; Wessels 1995) and additional radioisotope dating (Cs‐137 and Pb‐210; Jeter 2000). In 2010, sediment layers corresponding to the years 2000–2009 and 1965–1974 were sieved through a 224‐*μ*m mesh and obtained propagules inoculated in jars (20 per 100 mL) filled with a mix of 50% distilled tap water and 50% filtered (Whatman GF 92 prefilter and membrane filter 0.45 *μ*m) pond water (mesotrophic, no fish population; Botanical Garden, Frankfurt, Germany). The propagules from both time periods were not checked to see whether they contained viable eggs, nor for the number of eggs. The propagules were directly exposed to hatching stimuli, that is, light (16:8 L:D), elevated temperature (19 ± 1°C), and oxygen during time of extraction (Vandekerkhove et al. 2005). Each jar was checked daily for hatchlings over 3 weeks. Medium was renewed after 7 days. First hatchlings occurred at day three after incubation; the latest appeared at day nine. Within 24 h after hatching neonates were individually placed in 100 mL jars filled with ADaM (Aachener Daphnien Medium; Klüttgen et al. 1994) and kept under same conditions as the propagules. Every other day juveniles were fed with a suspension of the green algae *Acutodesmus obliquus,* and medium was renewed once a week. The species status was determined through Sanger sequencing of the mitochondrial 12S gene (Taylor et al. 1996) and nuclear genotyping using microsatellite loci (following protocols in Thielsch et al. 2009). In total, we kept 10 clonal lineages from 2000 to 2009 and eight lineages from 1965 to 1974 of *D. galeata* for more than 1 year under the described standardized laboratory conditions (16:8 L:D, 19 ± 1°C).

### Experimental setup – flow‐through system and temperature conditions

In October 2011, we conducted a common garden experiment comparing clonal lineages from 2000 to 2009 (thereafter named “recent”) with lineages from 1965 to 1974 (“historical”). Key life‐history parameters were measured under standardized laboratory conditions at three different temperatures. The temperatures were set at 22, 25, and 27°C. The lowest temperature regime (22°C) is most common in upper water layers during summer in Lake Constance, while the medium test temperature (25°C) is the current maximum during warm summer months (KLIWA, 2011). The highest test temperature (27°C) represented a slightly increased and more stressfull thermal regime, which is to be expected based on current climate models (IPCC, 2014). For the experiment, three identical climate chambers were equipped each with a 400‐L container filled with ADaM and holding individual tubes in a flow‐through system (Stich and Lampert 1984; Lampert et al. 1988). In this system, a peristaltic pump supplies a constant *A. obliquus* suspension into the experimental tubes (100 mL) with a mesh (200 *μ*m diameter) at the bottom. The flow‐through system provides a continuous unilateral flow through the tube, thus preventing chemical signaling between clonal lineages kept in different tubes positioned in the same container. Flow‐through rates (0.72 L per day & tube) were checked daily to avoid differences between climate chambers and tubes. Additionally, tube location in the container was changed daily in a random manner. Every 24 h each system was supplied with a fresh food suspension by refilling the 40‐L glass vessel connected to the peristaltic pump, with centrifuged algae resuspended in ADaM at a concentration of 1 mg·C·L^−1^. All chambers were supplied with the same solution at the same time each day to avoid potential room differences. The above‐mentioned carbon concentration largely exceeds the requirements for *D. galeata* and the measured available carbon in Lake Constance seston in summer (around 0.5 mg·C·L^−1^; Stich and Lampert 1981). A sufficient food supply is particularly necessary at higher test temperatures, as energy loss due to increased metabolic rates at higher temperatures can only be compensated with a higher carbon uptake (Giebelhausen and Lampert 2001; McFeeters and Frost 2011).

### Experimental setup – test animals

In pilot studies, we observed that acclimatization to experimental conditions is necessary to prevent physiological responses merely due to acute stress (Paul et al. 2004; Seidl et al. 2005), which interfere with phenotypic changes in response to thermal micro‐evolution (N. Henning‐Lucass, unpublished data). In order to acclimatize our test animals prior to the experiment, we introduced three “mother individuals” for each clonal lineage into each of the three climate chambers (Fig. S1). These mother individuals originated from our stock cultures and were born under standard laboratory conditions (19°C). Per clonal lineage, nine mother individuals (three per temperature regime) were taken as neonates from the second clutch of one mother from the third generation of our stock cultures. Therefore, we decreased interindividual variances and reduced maternal effects (Mousseau and Fox 1998). At the start of the experiment, neonates from the second clutch of the most reproductive mother individual per temperature regime were introduced in the flow‐through system within 12 h after birth, for three successive days. We chose a density of four individuals of the same clonal lineage per 100‐mL tube. In an additional experiment, we found no significant density effects (in tested life‐history traits) from one up to four individuals per tube (N. Henning‐Lucass, unpublished data). Mother individuals of three historical lineages were not able to produce the required number of four neonates within the 12‐h time frame. The experiment was thus performed with ten recent clonal lineages and five historical lineages per temperature regime.

### Variation in life‐history traits

At least six neonates per clonal lineage and born within the 12‐h time frame were not introduced into the experiment and instead conserved in ethanol to determine the mean neonate body size per clonal lineage at start of experiment. During the experiment, the following six life‐history traits were recorded, as they were shown to represent fitness estimates and vary under different environmental conditions (de Meester 1994; Lampert and Trubetskova 1996; Adrian et al. 2006): time to first reproduction (day), size at first reproduction (mm), size of first clutch (number of neonates per female), size of neonates (mm), survival rate (%), and somatic growth rate (SGR) (mm·day^−1^). Experimental tubes were checked every 24 h to control for neonates released from the brood pouch. As all individuals of one clonal lineage were inoculated in the same tube, we were not able to determine clutch size for each individual. In addition, although the females of one clonal lineage are genetically identical and exposed to the same conditions, not all females gave birth simultaneously. Therefore, we counted the neonates in the tube and in addition the unborn neonates in the carapace of all females of one tube to determine the average size of the first clutch for each tested clone. To capture the size of the females and the amount of unborn neonates in the carapace, females and neonates of each tube were transferred into ethanol after the first neonates were released from the brood pouch, and later photographed (Nikon SMZ 1500, Nikon GmbH, Düsseldorf, Germany) with NIS‐Elements 3.2 software. The size of the neonates as well as the size of mature females (Lampert 1993) was measured according to Flossner and Kraus (1986) from head to spine basis. The SGR was calculated using the formula [ln(*S*_*t*_) − ln(*S*_0_)] *t* ^−1^ (Wacker and von Elert 2001; Boersma and Kreutzer 2002), where *S*_0_ is the mean body size at start of the experiment of each clonal lineage and *S*_*t*_ the mean body size of the matured females of one clonal lineage, divided by the time to first reproduction (*t*) in days. For further analysis, the average value of the four individuals per experimental tube was calculated for each life‐history trait to estimate the clonal response at each temperature regime. Thus, the individuals of one tube were not used as independent replicates and for each clonal lineage and temperature one value was obtained. Survival rate was defined as the proportion of females surviving until release of the first clutch. Altogether we tested 15 tubes per climate chamber and reached a sample size of 45 tubes in total.

### Statistical analyses

Statistical analyses were performed with IBM SPSS Statistics 22 (IBM Corp., Armonk, NY). We used a multivariate general linear model to test for the effect of temperature, time period from which the clonal lineage was derived, and the interaction of both for all fitness variables. Subsequently, post hoc tests (Fisher's least significant difference) were performed to compare measured traits between two temperature treatments across the same set of clones. Additionally, we compared the sets of clones from different time periods (historical vs. recent) within each temperature regime using independent *t*‐tests in case of normally distributed data and Mann–Whitney *U*‐tests for not normally distributed data. To test for normality, we used the Lilliefors‐corrected Kolmogorov–Smirnov test. Alpha level was ≤0.05 for all tests. Figures were created with SigmaPlot 13.0.

## Results

**Figure 1:**
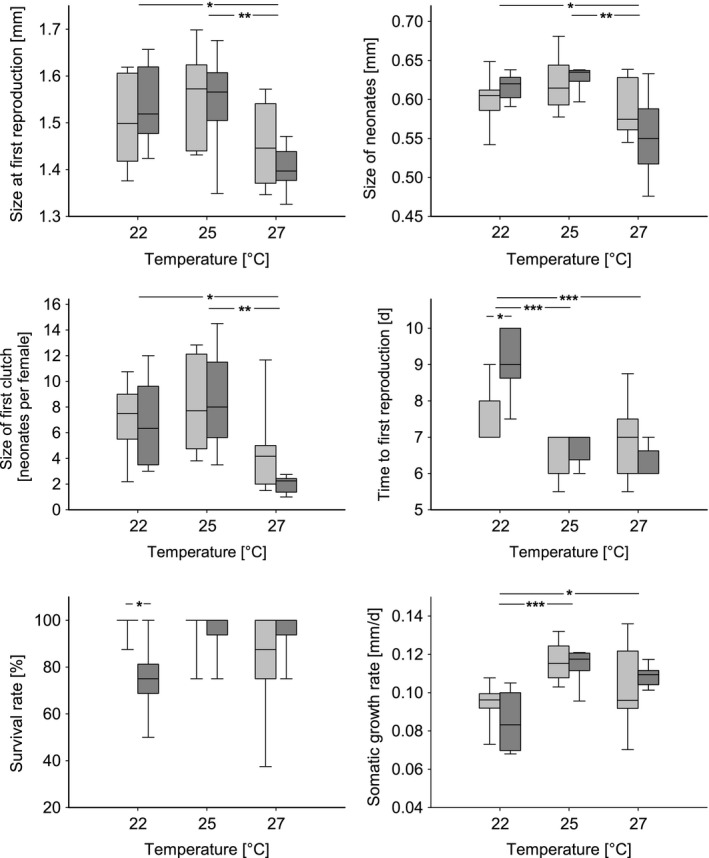
Boxplots showing significant temperature effects (pooled for clones from different time periods), as well as “temperature × clonal age” interactions for measured life‐history traits. Given are medians (black bars), first and third quartiles (box), as well as the highest and lowest value of the data (T‐bars). “Recent” clonal lineages (2000–2009) are shaded in light gray; “historical” lineages (1965–1974) are shaded in dark gray. Asterisks show significance levels (***P < 0.001; **P < 0.01; *P < 0.05).

Temperature had a significant effect on five of six measured life‐history traits (Table 1). Both sets of clones (i.e., historical and recent lineages pooled) showed plastic changes based on the thermal regime in size at first reproduction, size of neonates, size of first clutch, time to first reproduction, and somatic growth rate (Table 2, Fig. 1). Females of the 27°C treatment reproduced at a smaller size than females kept at 22°C and at 25°C. Neonate size was also smaller in the 27°C treatment than in 22 and 25°C. Furthermore, the size of first clutch was reduced in 27°C compared to 22 and 25°C. Both sets of clones responded to elevated water temperatures with a decrease in time to first reproduction and an increased somatic growth rate. Females in 22°C released the neonates from the brood pouch later compared to the 25 and 27°C treatment. Accordingly, the somatic growth rate was lowest in 22°C compared to 25 and 27°C.

**Table 1:** Multivariate general linear model testing for the effect of temperature, time period from which clonal lineage was derived and the interaction of both (Temperature × clonal age) on size at first reproduction (mm), size of neonates (mm), size of first clutch (neonates per female), time to first reproduction (day), survival rate (%), and somatic growth rate (SGR) (mm·day^−1^). Post hoc tests (Fisher's least significant difference, LSD) additionally tested for differences between temperature regimes. Significant *P*‐values (*P* < 0.05) are highlighted in bold

	*N*	df	*F*	*P*	*P* (LSD)		
					22°C vs 25°C	25°C vs 27°C	22°C vs 27°C
Size at first reproduction	45						
Temperature		2	6.255	**0.004**	0.264	**0.001**	**0.027**
Clonal age		1	0.105	0.748			
Temperature × clonal age		2	0.690	0.508			
Size neonates	45						
Temperature		2	7.597	**0.002**	0.188	**0.001**	**0.033**
Clonal age		1	0.072	0.789			
Temperature × clonal age		2	1.614	0.212			
Size of first clutch	45						
Temperature		2	5.935	**0.006**	0.341	**0.003**	**0.037**
Clonal age		1	0.491	0.488			
Temperature × clonal age		2	0.830	0.444			
Time to first reproduction	45						
Temperature		2	17.311	**<0.001**	**<0.001**	0.695	**<0.001**
Clonal age		1	0.832	0.367			
Temperature × clonal age		2	3.745	**0.033**			
Survival rate	45						
Temperature		2	1.187	0.316	0.393	0.092	0.393
Clonal age		1	0.248	0.621			
Temperature × clonal age		2	4.720	**0.015**			
Somatic growth rate	45						
Temperature		2	9.059	**0.001**	**<0.001**	0.070	**0.021**
Clonal age		1	0.098	0.756			
Temperature × clonal age		2	0.694	0.505			

**Table 2:** Means and standard errors given for life‐history traits of *Daphnia galeata* clones that differed significantly between temperature regimes

	22°C	25°C	27°C
Size at first reproduction (mm)	1.517 ± 0.024	1.556 ± 0.027	1.437 ± 0.020
Size neonates (mm)	0.605 ± 0.009	0.623 ± 0.009	0.575 ± 0.012
Size of first clutch (neonates per female)	6.86 ± 0.83	8.17 ± 0.94	3.92 ± 1.07
Time to first reproduction (day)	8.23 ± 0.29	6.60 ± 0.16	6.73 ± 0.29
Somatic growth rate (mm·day^−1^)	0.091 ± 0.004	0.116 ± 0.003	0.105 ± 0.005

Although we found no significant difference between sets of clones from different time periods across temperature treatments (Table 1), we observed significant “temperature × clonal age” interactions for time to first reproduction and survival rate (Table 1). Additional tests comparing the clonal lineages from the two tested time periods within each temperature regime revealed differences between historical and recent clonal lineages at 22°C. Recent clones exhibited a shorter time to first reproduction (*t* (12) = −2.491, *P* = 0.027), as well as a higher survival rate (*U* (12) = −2.659, *P* = 0.028), compared to the historical clones within the 22°C treatment (Fig. 1).

## Discussion

Our aim was to assess the level of adaptation of *D. galeata* to increased water temperatures over the last few decades. Comparing the adaptive response of clonal lineages from two different time periods, we hypothesized that recent clonal lineages would perform better under elevated water temperatures. Our results showed a general effect of increased temperatures on measured life‐history traits and further provided indication for a micro‐evolutionary response in measured life‐history traits.

### Life‐history responses to temperature in *D. galeata*

#### Size at maturity

In our experiment, we found that temperature affected all measured life‐history traits except for survival rate (Table 1). The temperature–size rule (Atkinson 1994) predicts a negative correlation between rearing temperature and size at first reproduction. Individuals in colder environments grow slower but mature at a larger body size, while the opposite is true for individuals of warmer environments, as it has been shown for a wide range of ectotherms, including zooplankton (Daufresne et al. 2009). This corresponds to our findings, with smaller females at 27°C compared to 22 and 25°C (Fig. 1). Our experiment revealed a tendency for highest values at 25°C (Fig. 1), potentially indicating an asymmetric‐shaped thermal response (Mitchell et al. 2004), which represents a rather general pattern in poikilotherms. The reaction norms for this thermal response are characterized by a gradual performance increase with higher temperatures, followed by a rapid decline after the upper thermal limit is reached (see also Kingsolver 2009). Khan and Khan (2008) attributed the observed differences in body size to thermal constraints operating as confounding factors on growth at higher temperatures. The authors associated an increased metabolism and involved energy expenditure at high temperatures with a decrease in *Daphnia* body size. This temperature‐enhanced activity might serve as an explanation for smallest females recorded at 27°C, assuming that higher energy demands could not be compensated with elevated food levels (McKee and Ebert 1996) or food quality (McFeeters and Frost 2011) and resulted in growth limitations (Stich and Lampert 1984).

Changes in body size are associated with morphological and physiological alterations, which may have a severe impact on various fitness‐related traits. For example, body size influences the reproductive success, as larger animals were shown to reach higher net reproductive rates (“Bigger is better” – hypothesis, Kingsolver and Huey 2008). In *Daphnia,* body size additionally affects predation risk (Kerfoot and Sih 1987) and competitive ability (“size‐efficiency hypothesis” Gliwicz 1990), which results in consequences on the community level with presumable impacts on ecosystem dynamics.

#### Neonate size and size of first clutch

In view of the above‐mentioned size‐related reproductive success, differences in maternal body size might also explain observed differences in neonate and clutch size. Further analyses revealed a correlation between maternal and neonate size (Pearson: *r*_(44)_ = 0.533, *P* < 0.001) as well as clutch size (*r*_(44)_ = 0.644, *P* < 0.001). This is in line with earlier studies on *Daphnia* species, where larger individuals produced bigger and more eggs (Glazier 1992; Lampert 1993). Compared to 22 and 25°C, neonate body length and the number of neonates per female decreased significantly at 27°C (Fig. 1), supporting the “bigger is better” – hypothesis (Kingsolver and Huey 2008) with the smallest females at 27°C.

#### Somatic growth rate and time to first reproduction

Warmer environments increased somatic growth rates at 25 and 27°C (Fig. 1). Accelerated growth and an accompanied earlier reproduction at elevated water temperatures is probably also a result of the increased metabolism that permits a rapid maturation and faster egg development (Stich and Lampert 1981). A smaller maturation threshold and earlier onset of reproduction presumably result in higher intrinsic rates of population increase (Kingsolver and Huey 2008; Harney 2013). As our experiment was terminated after the first clutch, we were unfortunately not able to measure and compare the overall reproductive potential between tested clones.

Altogether, the temperature of maximum response is approximately at 25°C (see also Mitchell et al. 2004). Test animals in the 22°C treatment had a reduced somatic growth rate and accordingly delayed reproduction, but nevertheless showed high life‐history trait values for body size and clutch size, slightly lower than those at 25°C. Animals of the 27°C treatment had, corresponding to the warm water temperature, an accelerated growth and early reproduction but lowest values for other measured life‐history traits.

### Temperature induced life‐history responses in relation to clonal age

Besides the above‐mentioned phenotypic plastic response to a gradient in temperature, the comparison of life‐history traits between clones from different time periods might also indicate a micro‐evolutionary response (Childs et al. 2016). “Temperature × clonal age” interactions were found with recent and historical clonal lineages differing in time to first reproduction and survival rate within the 22°C treatment. Recent clones reproduced earlier and had a higher survival rate compared to historical clones (Fig. 1). However, we found no significant difference in recorded life‐history traits between clonal lineages from different time periods at 25 and 27°C. This contradicts our expectation that clones differ at highest test temperatures, as recent clones witnessed increasing temperatures due to climatic changes during the last decades and consequently should have adapted to higher temperatures and perform better at these conditions. However, although maximum temperatures above 25°C are now common during warm summer months in the upper water layers of Lake Constance (KLIWA, 2011), these temperatures can still be considered as extreme for a species inhabiting a temperate lake (see also Moore et al. 1996). Although field observations and laboratory experiments (Stich and Lampert 1981, 1984) have shown that metabolic advantages in *D. galeata* through vertical migration are unlikely and the species principally occurs only at surface layers, migration through the water column remains a potential thermal avoidance behavior (see also Lagerspetz 2000) to elude extreme surface temperatures. Therefore, *Daphnia* might rarely be exposed to these temperatures under natural conditions and the higher test temperatures in our experiment may have elicited a stress response (Yampolsky et al. 2014), which might have obscured fine‐tuned responses of potentially better adapted clones. Recent clones might exhibit a better performance at 22°C, as they might be better adapted to the slightly increased mean summer temperatures (historical: 18–20°C, recent: 20–22°C), which *Daphnia* currently experiences during longer stretches of time in upper water layers from May to September. Thermal adaptation to current summer temperatures might explain differences between historical and recent clones within the 22°C treatment. If true, recorded phenotypic variance under transplant experimental conditions suggests micro‐evolutionary changes.

A highly variable environment imposes a strong selection pressure, to which populations or communities might respond by changes in species or genotype composition (Plard et al. 2016; Rees et al. 2016). Van Doorslaer et al. (2009b) artificially introduced putatively better adapted migrants in their mesocosm experiments, mimicking bird‐mediated migration events in *Daphnia* (Green and Figuerola 2005). They found a shift in the genetic composition of the mesocosm populations, thus evidencing a successful establishment of migrants. Through an extensive genotyping project of the Lake Constance *Daphnia* at different time periods, Brede (2008) and Herrmann (2010) were able to show that the historical population has not been replaced through migrants from other locations, at least not during our tested time period. Furthermore, hybridization can be excluded as a potential explanation for differences between our sets of clones, as we performed microsatellite analysis using multiple genetic markers to test for occurrence of mitochondrial introgression (Brede et al. 2009). A change in life‐history trait values, as a response to natural selection, might prevent population sorting and rapid micro‐evolutionary responses might reduce vulnerability for local invasions and replacements (Urban et al. 2008).

### Limitations of life‐history experiments

Earlier studies recorded differences in (inter)clonal responsiveness due to genetic and phenotypic variation (e.g., Boersma et al. 1999) and showed that clonal variability often enabled a response to environmental changes (Mitchell et al. 2004). Additionally, establishment and maintenance of clonal lineages in the laboratory might cause artificial selection. This bias is inherent to laboratory studies using laboratory stocks and might result in reduced genetic variation and evolutionary potential. In our experiment, genotyping of laboratory‐hatched and wild populations showed no significant differences in the genetic variability (Brede et al. 2007). However, we were not able to include all clonal lineages established in the laboratory in our experiments. As stated in the methods, in our preacclimation procedure we eliminated three historical clones due to the artificially set fecundity threshold of four neonates. The experiment was thus performed with only five of eight historical clonal lineages, thereby creating a potential bias. Excluding those less fecund lineages, which already might suggest a reduced fitness of historical lineages, potentially prevented us from recording more prominent differences in life‐history traits between clones, even at higher test temperatures. Additionally, another source of bias that may confound the comparison of historical and recent clones hatched from dormant stages is the presumably lower hatching rate of older eggs, which is common in resurrection studies. Although the experiment was based on laboratory cultures, founded from natural populations, the true natural variation might not be represented due to artificial selection and experimental limitations under laboratory conditions.

Further experiments are necessary to expand the lower test temperature range, including 18 and 20°C as temperature regimes, to cover the average summer temperatures in upper water layers during historical time periods and test for potentially better adapted historical clonal lineages. The upper limit of tested temperatures, however, need not be exceeded, as these were shown to be equally suboptimal for clones from both time periods. In order to enhance the statistical power a higher number of clonal lineages from both time periods would be necessary, as well as replicate tubes within each temperature treatment for each clonal lineage to examine potential clonal variation in thermal response.

## Conclusion

We showed that temperature had a significant effect on various life‐history traits of *D. galeata* and we were able to determine the temperature of maximum response (i.e., optimum temperature) at 25°C in our experiment, which is in accordance with earlier studies (e.g., Mitchell et al. 2004). However, we could not find any indication for a better performance of recent versus historical clones at increased temperature regimes (25 and 27°C), as originally hypothesized. Yet, we revealed a reduced time to first reproduction and a higher survival rate for recent compared to historical clonal lineages within the 22°C treatment. The observed phenotypic differences may suggest a micro‐evolutionary response based on thermal adaptation of recent clones to slightly increased summer temperatures (see also van Doorslaer et al. 2010). However, our findings do not allow for an explicit conclusion on the adaptive nature as experimental limitations have to be considered.

## Conflict of Interest

None Declared.

## Supporting information


**Figure S1.** Scheme to illustrate the culturing and acclimatisation of test animals.
