# Supplementary material for: Phenotypic plasticity in life‐history traits of Daphnia galeata in response to temperature – a comparison across clonal lineages separated in time
Source: Ecol Evol. 2016 Feb 22;6(4):881–91. doi: 10.1002/ece3.1924 (PMC4761779; doi:10.1002/ece3.1924)
Supplement: Supplementary file 1 — Figure S1. Scheme to illustrate the culturing and acclimatisation of test animals. [file ECE3-6-0881-s001.pdf]

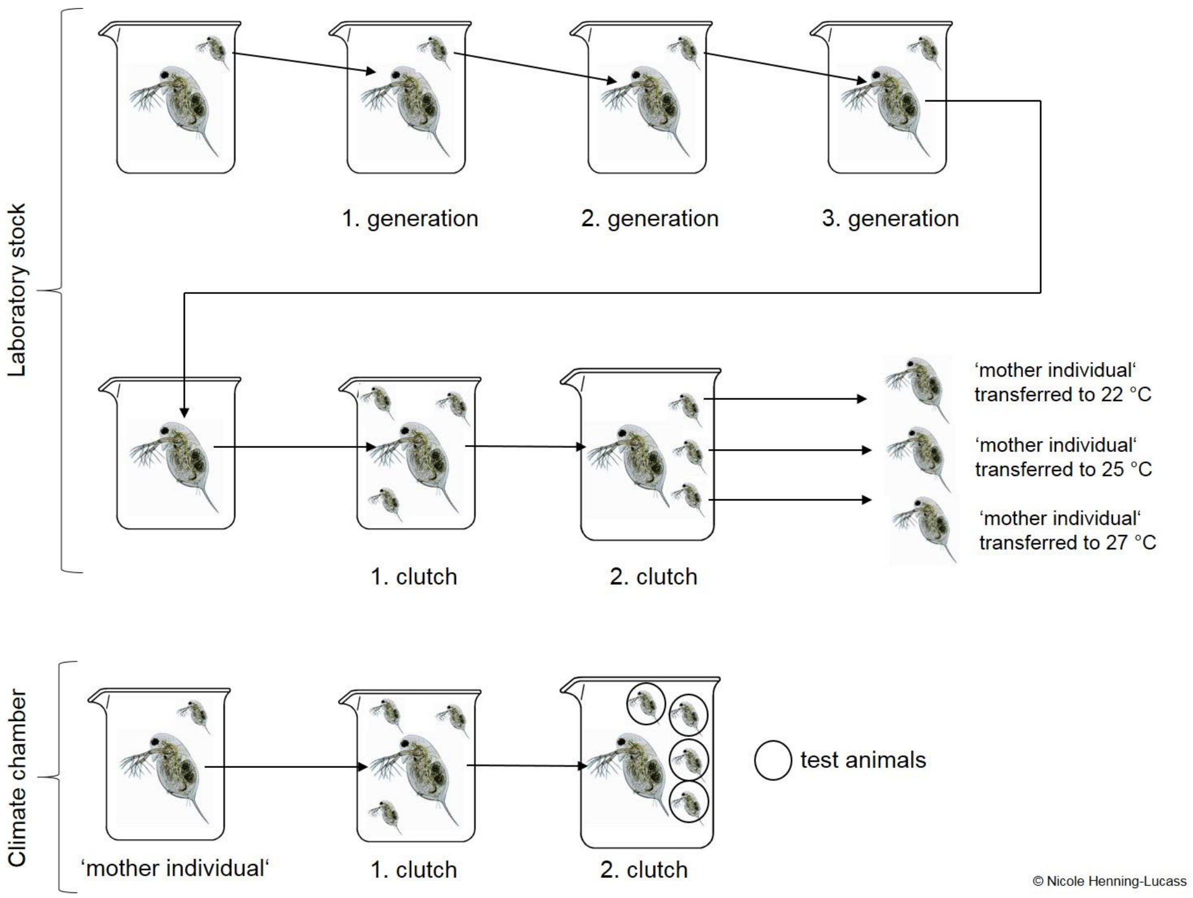

Fig. S1 Scheme to illustrate the culturing and acclimatisation of test animals. In order to reduce maternal effects, the ‘mother individuals’ of our test animals were taken from the second clutch of one female (per clonal lineage), which originated from the third generation of our laboratory stock cultures (19 °C). ‘Mother individuals’ were transferred as neonates into the climate chambers (22 °C, 25 °C or 27 °C). Four neonates (per clone) from the second clutch of a mother individual were introduced in the flow-through system as test animals.
